# Severe Bacteremia Caused by *Clostridium butyricum* Following Endoscopic Ultrasound‐Guided Peripancreatic Fluid Drainage for Walled‐off Necrosis: A Case Report

**DOI:** 10.1002/deo2.70325

**Published:** 2026-04-11

**Authors:** Koichi Soga, Ou Takagi, Haruka Kato, Hiroki Maeda, Yuki Soma, Masaru Kuwada, Yo Fujimoto, Ryosaku Shirahashi, Ikuhiro Kobori, Masaya Tamano

**Affiliations:** ^1^ Department of Gastroenterology Dokkyo Medical University Saitama Medical Center Saitama Japan

**Keywords:** *Clostridium butyricum*, endoscopic ultrasound‐guided peripancreatic fluid drainage, plastic stent dislodgement, probiotic‐related bacteremia, walled‐off necrosis

## Abstract

We report a rare case of bacteremia caused by *Clostridium butyricum* following endoscopic ultrasound‐guided peripancreatic fluid drainage (EUS‐PFD) for walled‐off necrosis (WON) in a patient with alcoholic chronic pancreatitis. The patient underwent EUS‐PFD using a plastic stent (PS) for WON, initially with a good response. However, dislodgement of the PS resulted in closure and secondary cavity infection. The patient subsequently developed sepsis and multiorgan failure. Blood cultures from arterial and venous sources confirmed *C. butyricum*, consistent with the patient's oral intake of *C. butyricum* MIYAIRI 588 (CBM 588). Intensive care, including repeated EUS‐PFD and antibiotic administration, led to recovery. Although CBM 588, a *C. butyricum agent*, remains a valuable probiotic, clinicians must exercise caution when prescribing it to patients with impaired mucosal barriers or those undergoing EUS‐guided drainage procedures. Close monitoring of stent placement, drainage efficacy, and individualized assessment of probiotic administration are essential to minimize the risk of systemic infections.

**Trial Registration**: The authors have confirmed clinical trial registration is not needed for this submission.

**Abbreviations**
ACP: alcoholic chronic pancreatitis;
CRP: C‐reactive protein;
CT: computed tomography;
DPPS: double‐pigtail plastic stents;
EUS‐PFD: endoscopic ultrasound‐guided peripancreatic fluid drainage;
ICU: intensive care unit;
LDH: lactate dehydrogenase;
PS: plastic stent;
WON: walled‐off necrosis.

## 1 Introduction

*Clostridium butyricum* is a strictly anaerobic, spore‐forming bacillus commonly found in the environment and has been found as a commensal organism in the gastrointestinal tracts of humans and animals [1]. *C. butyricum* MIYAIRI 588 (CBM 588) is a probiotic strain that is widely used in Japan and other parts of Asia for promoting gut health by producing butyrate, enhancing intestinal barrier function, and modulating immunity. CBM 588 is generally safe and has been used to prevent antibiotic‐associated diarrhea and *Clostridioides difficile* infection [2]. However, reports have raised concerns about bacteremia in patients with compromised mucosal barriers or undergoing interventional endoscopic procedures [3, 4].

We present a unique case of bacteremia with *C. butyricum* following endoscopic ultrasound‐guided peripancreatic fluid drainage (EUS‐PFD) in a patient with alcoholic chronic pancreatitis (ACP) and suboptimal adherence to dietary recommendations. The patient was taking CBM 588 orally three times per day. The patient developed sepsis and multiorgan failure, necessitating intensive care unit (ICU) management.

## 2 Case Report

**FIGURE 1:**
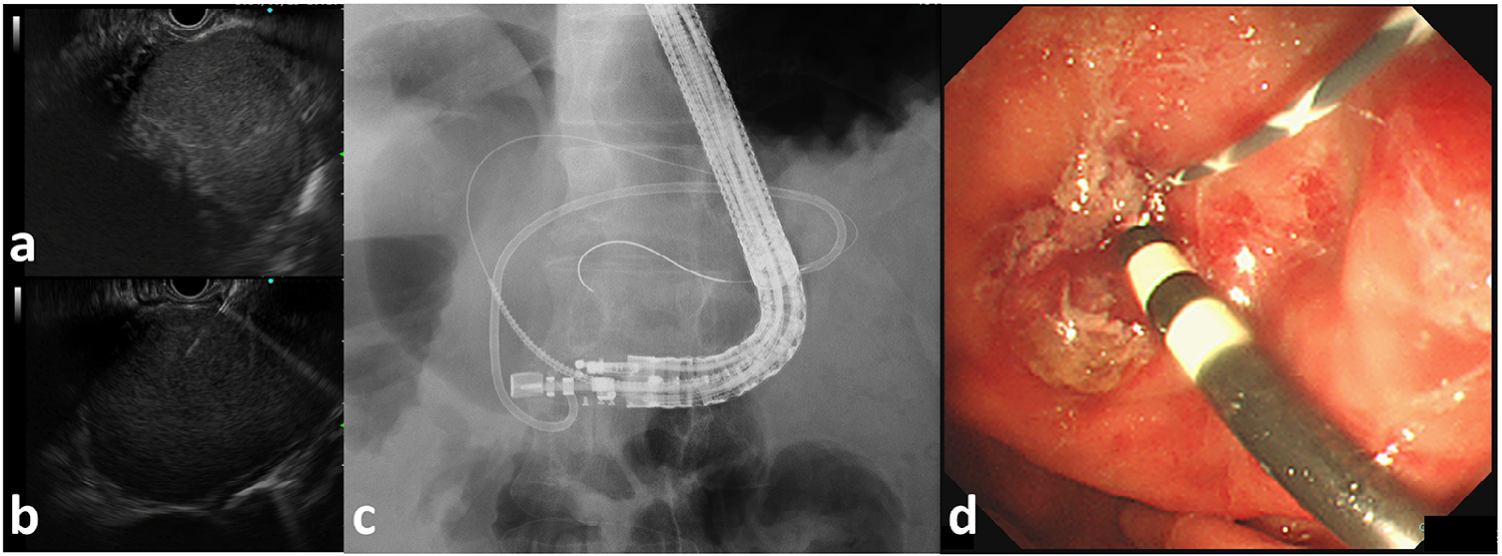
Endoscopic ultrasound‐guided peripancreatic fluid drainage (EUS‐PFD) using novel half‐double pigtail plastic stents (H‐DPPS, 7Fr × 10 cm, Piglet; Olympus, Tokyo, Japan). Two months before this presentation, EUS‐PFD was performed for walled‐off necrosis (WON) using the novel H‐DPPS. The procedure was uneventful, and the patient was discharged in stable condition. (a) Convex array EUS image showing an encapsulated WON adjacent to the stomach wall. (b) Real‐time EUS image demonstrating safe puncture of the necrotic cavity with a 19‐gauge fine‐needle aspiration (FNA) needle under direct visualization. (c) Fluoroscopic image obtained after placement of two guidewires: the first H‐DPPS was inserted, and tract dilation was performed over the second guidewire using a drill‐type dilator. (d) Endoscopic view showing deployment of H‐DPPS into the cyst cavity; the second guidewire remains visible alongside the first stent.

**FIGURE 2:**
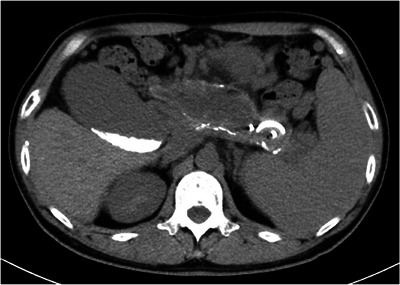
Follow‐up computed tomography (CT) image after endoscopic ultrasound‐guided peripancreatic fluid drainage (EUS‐PFD) using novel half‐double pigtail plastic stents (H‐DPPS, 7Fr × 10 cm, Piglet; Olympus, Tokyo, Japan). Non‐contrast CT performed after EUS‐PFD showed that the H‐DPPS was appropriately positioned within the walled‐off necrosis (WON) cavity. The size of the WON had decreased, indicating successful drainage and resolution of the collection.

**FIGURE 3:**
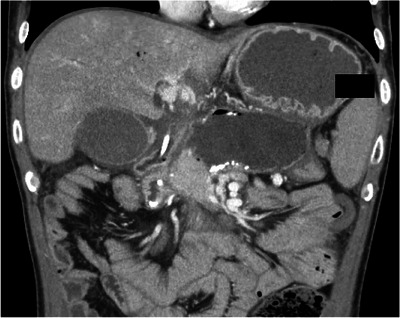
Coronal computed tomography (CT) image showing recurrence of walled‐off necrosis (WON) due to stent dislodgement and gastric outlet obstruction (GOO). The patient developed GOO associated with a worsening duodenal stricture secondary to alcoholic chronic pancreatitis (ACP), presenting with regurgitation of food residue and gastric fluid. The patient was readmitted with altered consciousness and severe abdominal pain. CT revealed dislodgement of the half‐double pigtail plastic stent (H‐DPPS, 7Fr × 10 cm, Piglet; Olympus, Tokyo, Japan) previously placed for EUS‐guided peripancreatic fluid drainage (EUS‐PFD), and the cyst cavity was markedly distended with fluid and gas.

A man in his 30s with a history of ACP was hospitalized for alcoholic acute pancreatitis 6 years prior. He continued drinking and developed severe acute pancreatitis during this period, requiring ICU treatment. Walled‐off necrosis (WON) was noted during recovery (Figure S1). Following discharge, the patient was followed as an outpatient for ACP for 5 years. Six months before the current presentation, he began complaining of abdominal distension. Computed tomography (CT) revealed enlargement of the preexisting WON (Figure S2). Two months prior, EUS‐PFD was performed using half‐double‐pigtail plastic stents (H‐DPPS; 7Fr X 10 cm; Piglet, Olympus, Tokyo, Japan). The procedure was uneventful, and the patient was discharged in stable condition (Figures 1 and 2, Video S1). Despite discharge instructions, the patient had poor dietary compliance. He was readmitted with altered consciousness and severe abdominal pain. CT showed dislodgement of the H‐DPPS and a distended cyst cavity containing fluid and gas, consistent with secondary infection (Figure 3). He was referred to our hospital for management of a severe infection. Upon arrival, his level of consciousness was assessed as E3V4M5 on the Glasgow coma scale, and vital signs revealed a fever of 38.5°C, a blood pressure of 68/– mmHg, and a regular pulse rate of 180 beats per minute. Because his mental status was progressively deteriorating and clinical findings were consistent with sepsis‐induced hemodynamic instability, norepinephrine infusion was initiated to maintain systemic circulation. Given his decreased level of consciousness, he was endotracheally intubated and placed on mechanical ventilation for respiratory support. Laboratory findings on admission are summarized in Table S1.

**FIGURE 4:**
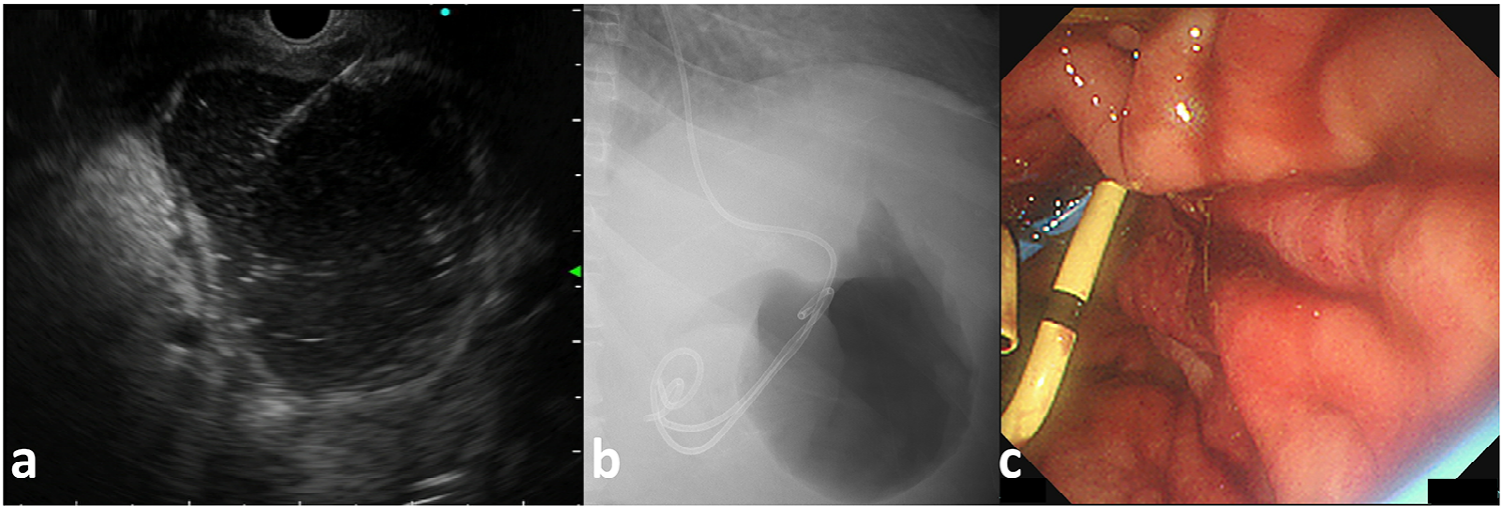
Emergency endoscopic ultrasound‐guided peripancreatic fluid drainage (EUS‐PFD) using a double pigtail plastic stent (DPPS; 7Fr × 7 cm, Through and Pass, Gadelius Medical, Tokyo, Japan) and an endoscopic nasobiliary drainage (ENBD) tube (6Fr, Flexima; Boston Scientific, Massachusetts, USA). (a) Convex array EUS image showing an encapsulated WON adjacent to the stomach wall. Real‐time EUS‐guided puncture of the necrotic cavity was safely performed using a 19‐gauge fine‐needle aspiration (FNA) needle under direct visualization. (b) Fluoroscopic image obtained after the placement of two guidewires. The first DPPS (7Fr × 7 cm, Through and Pass) and ENBD tube (6Fr, Flexima) were successfully inserted for internal and external drainage. (c) Endoscopic image showing the deployment of the DPPS into the cyst cavity during the procedure.

EUS‐PFD was reattempted using another DPPS (7Fr × 7 cm; Through and Pass, Gadelius Medical, Tokyo, Japan) and an endoscopic nasobiliary drainage tube for external drainage (6Fr, Flexima; Boston Scientific, Massachusetts, USA) (Figure 4). Empirical antibiotics were initiated. Despite successful intervention, the patient's condition deteriorated, developing septic shock and multiorgan failure, requiring ICU admission.*C. butyricum* was isolated from both arterial (one set) and venous (one set) blood cultures. Cultures of drained fluid/debris from the WON cavity did not yield *C. butyricum* but demonstrated a polymicrobial profile (Table S2). Together with the contrast‐enhanced CT findings suggestive of small‐bowel involvement (bowel wall edema and portal venous gas), we strongly suspected non‐occlusive mesenteric ischemia (NOMI) involving the small intestine (Video S1). The absence of *C. butyricum* in the abscess supports an alternative pathophysiologic mechanism—ischemia‐related mucosal injury with intestinal barrier disruption and subsequent bacterial translocation—by which the commensal organism may have entered the bloodstream. Thus, *C. butyricum* bacteremia was unlikely to have resulted from direct dissemination from the abscess itself. The patient had been taking CBM 588 at home. Antibiotic therapy was adjusted based on sensitivity results, and intensive supportive care was provided. The bacteremia resolved four days later, and the patient was gradually weaned off organ support and discharged 2 months later following the second EUS‐PFD (Figure S3).

## 3 Discussion

WON is a late complication of acute necrotizing pancreatitis, characterized by liquefied pancreatic necrosis encapsulated by a well‐defined, fibrous, and granulation tissue wall. Intervention for non‐infected WON is generally indicated when patients develop symptoms, such as abdominal pain, nausea, vomiting, or signs of infection [5]. In recent years, EUS‐PFD has emerged as a highly effective and minimally invasive strategy for managing WON. Several studies have demonstrated that this approach achieves favorable clinical outcomes while avoiding invasive surgical options [5, 6, 7]. EUS‐PFD is now standard; however, the risk of infection remains a major concern. Inadequate drainage due to stent dislodgement, food residue influx into the cyst cavity, and increased intraluminal pressure likely contributed to secondary infections in our case.

In the context of EUS‐PFD, the utility of DPPS has already been well‐established [8, 9]. A key theoretical advantage of the DPPS design is its circular terminal “pigtail” curvature, which acts as a mechanical lock to anchor the stent in both the gastrointestinal lumen and the cyst cavity. Even after the cyst cavity shrinks, only the coiled ends of the DPPS remain embedded, indicating that the retention mechanism continues to function even as fluid collection resolves. In contrast, the H‐DPPS used in our case exhibited a gentler curve at both ends, which may have been insufficient to securely anchor the stent within a large or deep cyst cavity. As a result, stent migration back into the gastrointestinal lumen may occur more readily, particularly in large or deep collections, where the cavity is not firmly confined.

In this case, blood cultures confirmed bacteremia caused by *C. butyricum*. However, we did not conduct a systematic literature search or a detailed investigation to substantiate this association. We hypothesized that the recurrence of WON formation and systemic inflammatory response may have precipitated hemodynamic compromise, leading to NOMI and subsequent *C. butyricum* bacteremia. Notably, abscess microbiology reflected a distinct polymicrobial process not dominated by this organism.

This microbiological discrepancy provides evidence against direct hematogenous dissemination from the abscess as the primary source of the bacteremia. Contrast‐enhanced CT findings suggestive of small‐bowel involvement alongside the absence of *C. butyricum* in the abscess indicate that ischemia‐related mucosal injury with intestinal barrier disruption and subsequent bacterial translocation may have facilitated entrance into the bloodstream.

We considered the possibility that regular intake of CBM588 may have contributed to or facilitated the development of *C. butyricum* bacteremia. CBM 588 is widely prescribed in Japan for the prevention of gastrointestinal infections and antibiotic‐associated diarrhea [2, 4]. Probiotic‐related bacteremia is rare; however, it predominantly affects hospitalized patients with significant comorbidities. Although considered safe, recent studies have documented rare cases of CBM 588‐associated bacteremia. Ishikawa et al. [3] reported three cases in Japan, mostly in immunocompromised patients or those with intestinal barrier injuries. Similarly, CBM 588 was identified in 0.08% of positive blood cultures, all genetically matched to probiotic strains [4].

Because the patient had been taking CBM 588 long term, this intake may have contributed to the development of *C. butyricum* bacteremia. However, beyond routine culture results, no further investigations (e.g., strain‐level identification or genetic analyses) were performed; therefore, we could not directly confirm a causal link or prove probiotic‐derived bacteremia. Nevertheless, prior reports have evaluated bacteremia potentially associated with CBM 588 administration, and our case underscores the need for careful indication assessment and judicious strain selection when prescribing probiotics, particularly in patients with high‐risk conditions such as impaired mucosal integrity, severe infection, or those undergoing interventional endoscopic procedures.

This case emphasizes four critical clinical implications: (1) Close postprocedural monitoring is vital after EUS‐guided drainage, particularly with respect to stent position and drainage function. (2) Individualized evaluation of probiotic administration should be performed, particularly in the context of mucosal injury, chronic inflammation, or ongoing infection. (3) From an endoscopic perspective, device selection for EUS‐related procedures requires careful consideration, and sensitive device selection is desirable in each case.

This case report represents a rare instance of *C. butyricum*‐associated bacteremia following EUS‐PFD. Clinicians should carefully evaluate probiotic use in patients with impaired mucosal integrity or in those undergoing interventional EUS procedures. Attention to stent selection and close postprocedural monitoring may help prevent similar complications.

## Author Contributions

**Koichi Soga**: conceptualization, data curation, formal analysis, investigation, methodology, project administration, resources, supervision, validation, visualization, writing of the original draft, writing the review, and editing. **Ou Takagi, Haruka Kato, Hiroki Maeda, Yuki Soma, Masaru Kuwada, Yo Fujimoto, Ryosaku Shirahashi, Ikuhiro Kobori, and Masaya Tamano**: writing the review and editing.

## Funding

The authors have nothing to report.

## Ethics Statement

The authors have nothing to report.

## Consent

Written consent for publication was obtained from the patient.

## Conflicts of Interest

The authors declare no conflicts of interest.

## Supporting information




**Figure S1**: Non‐contrast computed tomography (CT) image in a man with alcoholic chronic pancreatitis (ACP). A man in his 30s with a history of ACP was hospitalized for alcoholic acute pancreatitis 6 years prior. He continued drinking alcohol and later developed severe acute pancreatitis, which required intensive care. During recovery, walled‐off necrosis (WON) was identified around the pancreas.

**Figure S2**: Non‐contrast computed tomography (CT) image showing enlargement of the preexisting walled‐off necrosis (WON). Six months before presentation, the patient complained of progressive abdominal distension. Non‐contrast CT demonstrated marked enlargement of the previously identified WON around the pancreatic region.

**Figure S3**: Clinical course and temporal changes in laboratory parameters following endoscopic ultrasound‐guided peripancreatic fluid drainage (EUS‐PFD) in a patient with severe *Clostridium butyricum* (*C. butyricum*) bacteremia. This figure illustrates the clinical course and changes in key laboratory markers following EUS‐PFD in a patient with infected walled‐off necrosis and subsequent severe sepsis due to *C. butyricum*. The graph shows trends in the following laboratory parameters over 46 hospital days: white blood cell count (WBC, blue), platelet count (PLT, orange), C‐reactive protein (CRP, green), lactate dehydrogenase (LDH, red), and serum creatinine (Cre, purple). The patient experienced a rapid elevation of inflammatory markers (WBC, CRP, and LDH) and a sharp decrease in platelet count at admission, consistent with septic shock and multiorgan dysfunction. EUS‐PFD was performed on Day 1. Despite early intervention, the patient developed severe septicemia requiring endotracheal intubation, continuous renal replacement therapy (CRRT), and broad‐spectrum antimicrobial therapy including vancomycin (VCM), meropenem (MEPM), and sulbactam/ampicillin (SBT/ABPC). Gradual improvement was observed in laboratory data and hemodynamics, and the patient was discharged on Day 46.

**Table S1**: Admission laboratory parameters.

**Table S2**: Comprehensive culture results from arterial blood, venous blood, and abscess specimens.

**Video S1**: Coronal computed tomography image showing recurrence of walled‐off necrosis due to stent dislodgement and gastric outlet obstruction.
