# Supplementary figures and images for: Severe Bacteremia Caused by Clostridium butyricum Following Endoscopic Ultrasound‐Guided Peripancreatic Fluid Drainage for Walled‐off Necrosis: A Case Report
Source: DEN Open. 2026 Apr 11;6(1):e70325. doi: 10.1002/deo2.70325 (PMC13069354; doi:10.1002/deo2.70325)

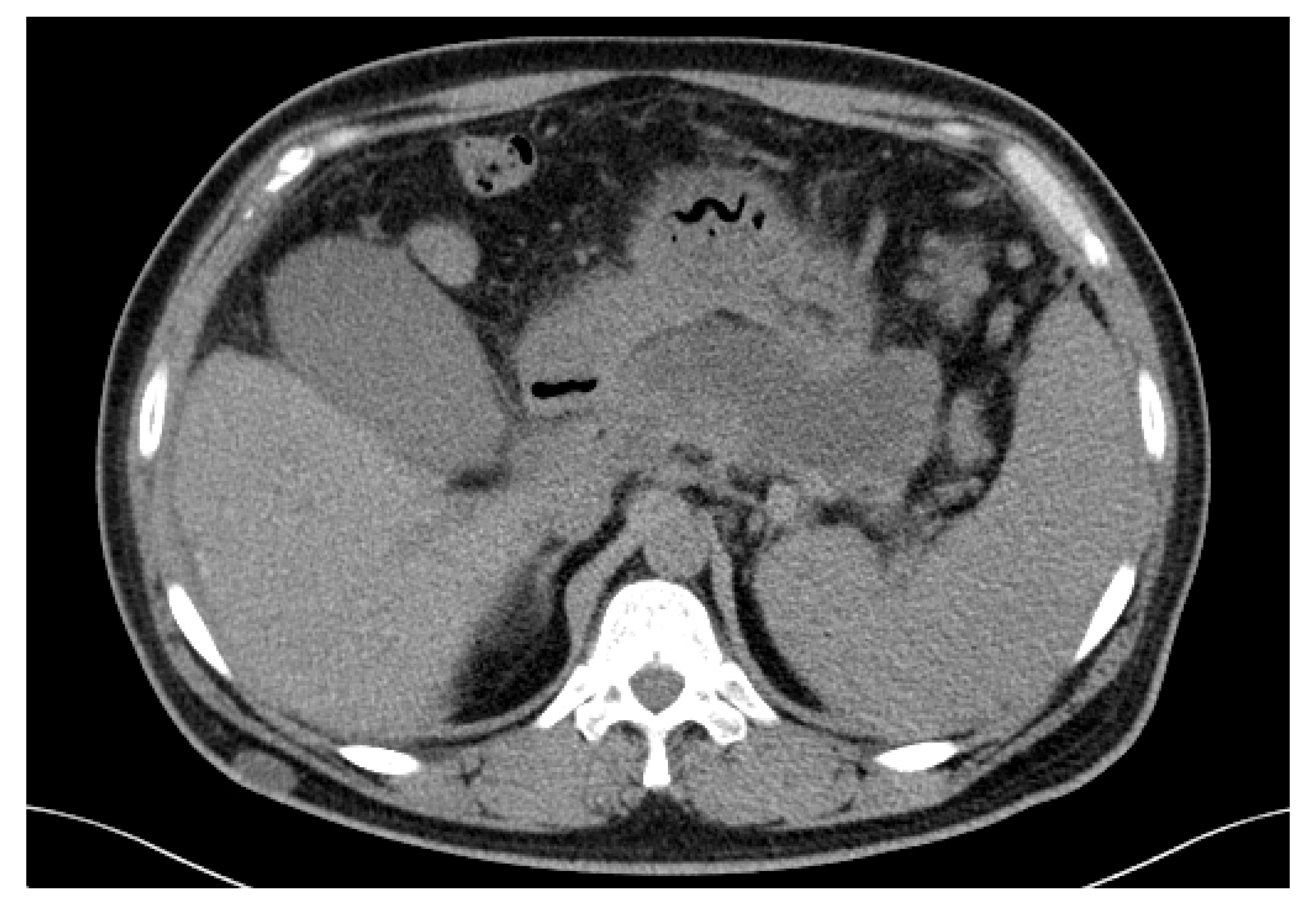

Supplement: Supplementary file 1 — Figure S1: Non‐contrast computed tomography (CT) image in a man with alcoholic chronic pancreatitis (ACP). A man in his 30s with a history of ACP was hospitalized for alcoholic acute pancreatitis 6 years prior. He continued drinking alcohol and later developed severe acute pancreatitis, which required intensive care. During recovery, walled‐off necrosis (WON) was identified around the pancreas. [file DEO2-6-e70325-s004.jpg]

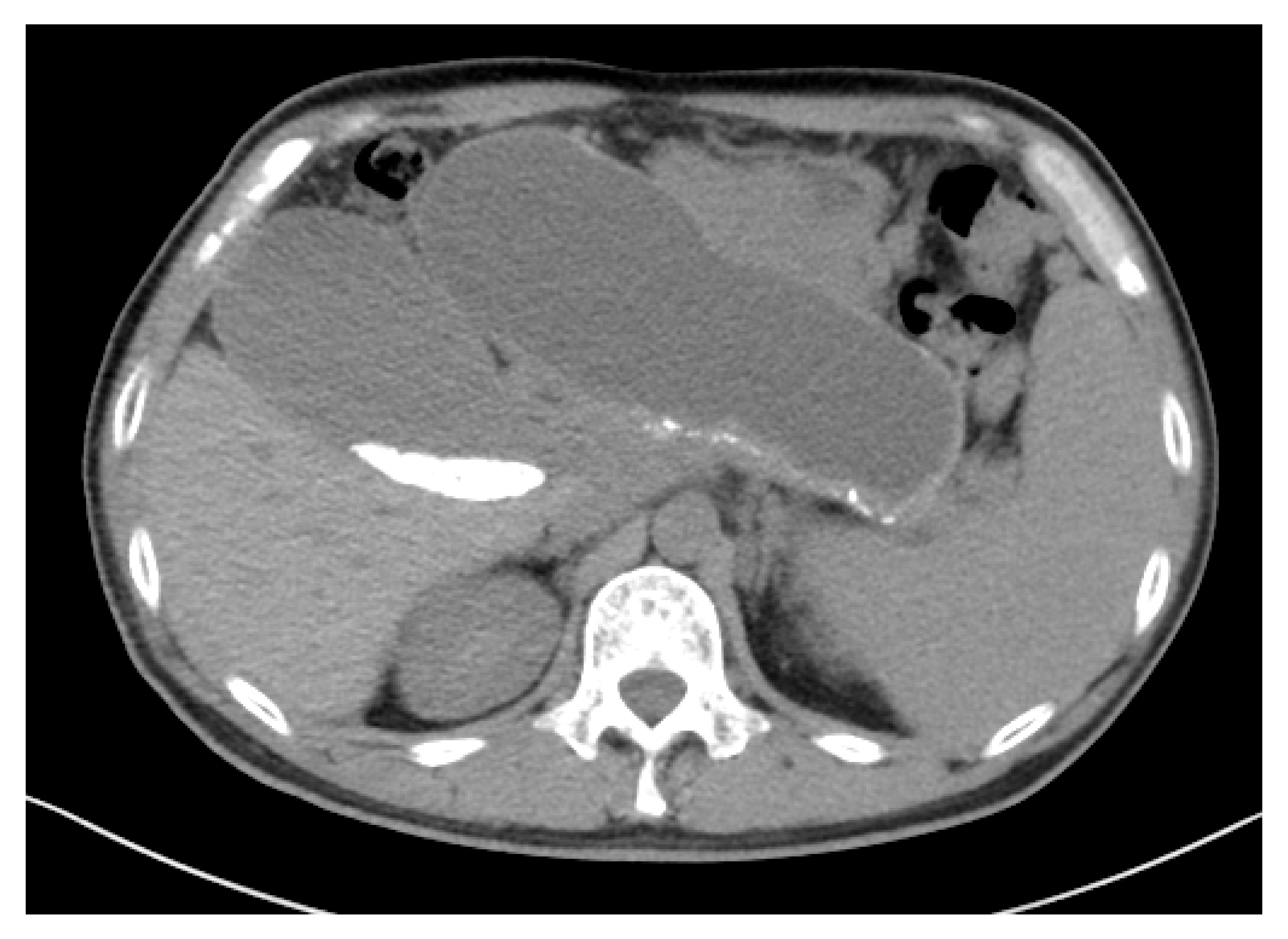

Supplement: Supplementary file 2 — Figure S2: Non‐contrast computed tomography (CT) image showing enlargement of the preexisting walled‐off necrosis (WON). Six months before presentation, the patient complained of progressive abdominal distension. Non‐contrast CT demonstrated marked enlargement of the previously identified WON around the pancreatic region. [file DEO2-6-e70325-s001.jpg]

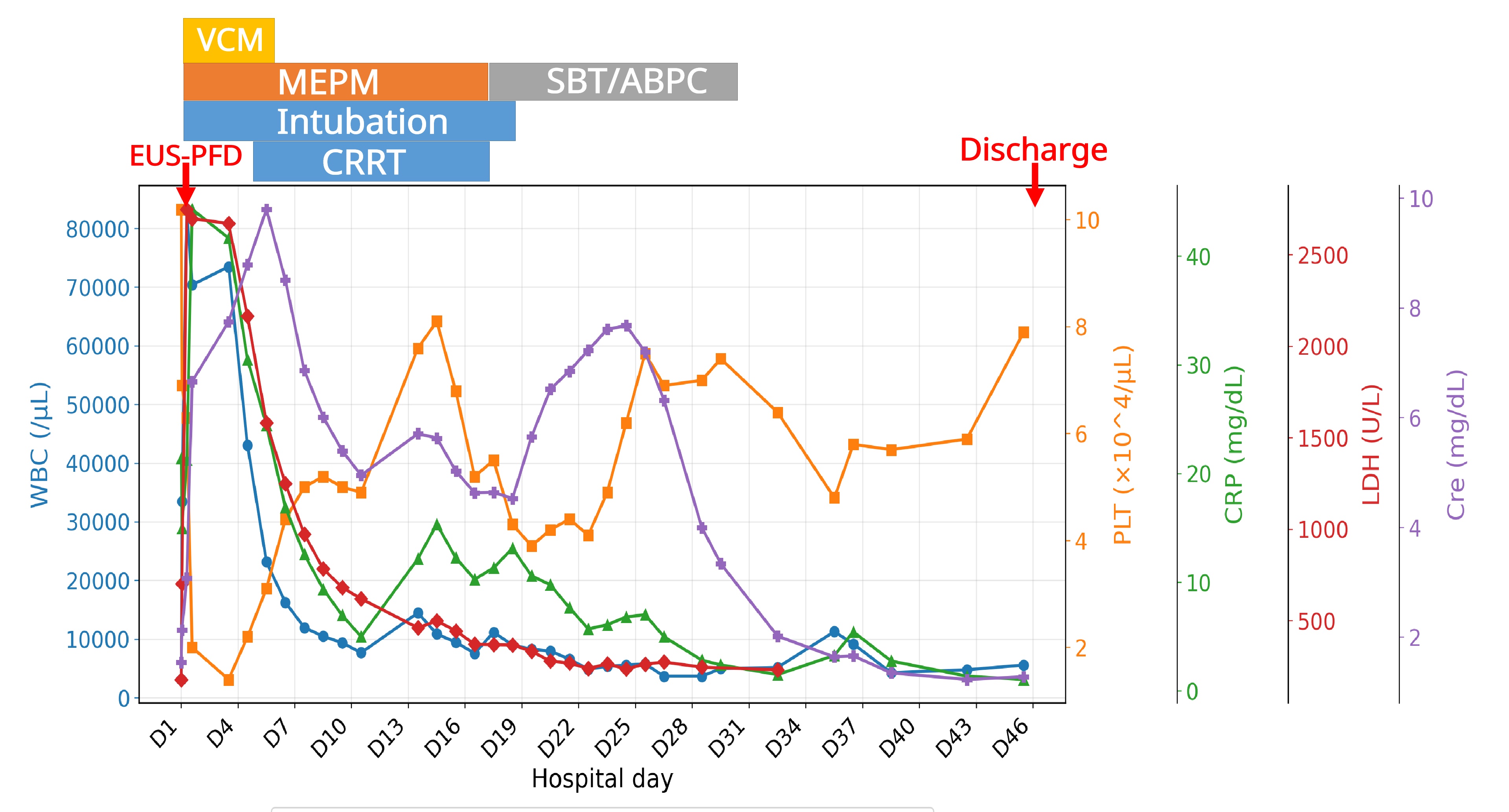

Supplement: Supplementary file 3 — Figure S3: Clinical course and temporal changes in laboratory parameters following endoscopic ultrasound‐guided peripancreatic fluid drainage (EUS‐PFD) in a patient with severe Clostridium butyricum (C. butyricum) bacteremia. This figure illustrates the clinical course and changes in key laboratory markers following EUS‐PFD in a patient with infected walled‐off necrosis and subsequent severe sepsis due to C. butyricum. The graph shows trends in the following laboratory parameters over 46 hospital days: white blood cell count (WBC, blue), platelet count (PLT, orange), C‐reactive protein (CRP, green), lactate dehydrogenase (LDH, red), and serum creatinine (Cre, purple). The patient experienced a rapid elevation of inflammatory markers (WBC, CRP, and LDH) and a sharp decrease in platelet count at admission, consistent with septic shock and multiorgan dysfunction. EUS‐PFD was performed on Day 1. Despite early intervention, the patient developed severe septicemia requiring endotracheal intubation, continuous renal replacement therapy (CRRT), and broad‐spectrum antimicrobial therapy including vancomycin (VCM), meropenem (MEPM), and sulbactam/ampicillin (SBT/ABPC). Gradual improvement was observed in laboratory data and hemodynamics, and the patient was discharged on Day 46. [file DEO2-6-e70325-s002.jpg]
