# Supplementary material for: Severe Bacteremia Caused by Clostridium butyricum Following Endoscopic Ultrasound‐Guided Peripancreatic Fluid Drainage for Walled‐off Necrosis: A Case Report
Source: DEN Open. 2026 Apr 11;6(1):e70325. doi: 10.1002/deo2.70325 (PMC13069354; doi:10.1002/deo2.70325)
Supplement: Supplementary file 4 — Table S1: Admission laboratory parameters. [file DEO2-6-e70325-s006.docx]

| **Complete Blood Count** |  |  |  | **Serum Biochemistry** |  |  |  | **Arterial Blood Gas Analysis (Intubation)** | |  |
| --- | --- | --- | --- | --- | --- | --- | --- | --- | --- | --- |
| White blood cell count | 83.3 | ×10^3/μL |  | Aspartate aminotransferase | 746 | U/L |  | Arterial pH | 7.339 |  |
| Segmented neutrophils | 62 | % |  | Alanine aminotransferase | 264 | U/L |  | Arterial pCO₂ (PaCO₂) | 31.9 | mmHg |
| Band neutrophils | 18 | % |  | Alkaline phosphatase | 354 | U/L |  | Arterial pO₂ (PaO₂) | 159.3 | mmHg |
| Lymphocytes | 13 | % |  | Lactate dehydrogenase | 2749 | U/L |  | Base excess (ECF) (BEecf) | -9 | mmol/L |
| Monocytes | 4 | % |  | Gamma-glutamyl transferase | 236 | U/L |  | Base excess (blood) (BEb) | -7.9 | mmol/L |
| Metamyelocytes | 3 | % |  | Total bilirubin | 1.72 | mg/dL |  | Bicarbonate (HCO₃⁻) | 16.8 | mmol/L |
| Red blood cell count | 4.21 | ×10^6/μL |  | Direct bilirubin | 0.78 | mg/dL |  | Total CO₂ (TCO₂) | 17.8 | mmol/L |
| Hemoglobin | 12.3 | g/dL |  | Indirect bilirubin | 0.94 | mg/dL |  | Standard bicarbonate (SBC) | 18.1 | mmol/L |
| Hematocrit | 36.3 | % |  | Total protein | 4.5 | g/dL |  | Oxygen saturation (calculated) (%sO₂c) | 96.4 | % |
| Platelet count | 63 | ×10^3/μL |  | Albumin | 2.43 | g/dL |  | Arterial lactate | 51.2 | mg/dL |
|  |  |  |  | Sodium | 138 | mmol/L |  |  |  |  |
| **Coagulation Profile** |  |  |  | Potassium | 4 | mmol/L |  |  |  |  |
| Fibrinogen | 67 | mg/dL |  | Chloride | 106 | mmol/L |  |  |  |  |
| D-dimer | 543.7 | µg/mL |  | Calcium | 7.6 | mg/dL |  |  |  |  |
| Antithrombin III | 30 | % |  | Magnesium | 1.7 | mg/dL |  |  |  |  |
| Fibrin/fibrinogen degradation products | >960.0 | µg/mL |  | Blood urea nitrogen | 29 | mg/dL |  |  |  |  |
| Prothrombin time | 27.8 | sec |  | Creatinine | 3.08 | mg/dL |  |  |  |  |
| PT activity | 16.6 | % |  | Uric acid | 8.1 | mg/dL |  |  |  |  |
|  |  |  |  | Glucose | 212 | mg/dL |  |  |  |  |
|  |  |  |  | C-reactive protein | 21.27 | mg/dL |  |  |  |  |
|  |  |  |  |  |  |  |  |  |  |  |

**Supporting Table S1 Laboratory Findings Upon Submission**
