# Supplementary material for: Severe Bacteremia Caused by Clostridium butyricum Following Endoscopic Ultrasound‐Guided Peripancreatic Fluid Drainage for Walled‐off Necrosis: A Case Report
Source: DEN Open. 2026 Apr 11;6(1):e70325. doi: 10.1002/deo2.70325 (PMC13069354; doi:10.1002/deo2.70325)
Supplement: Supplementary file 5 — Table S2: Comprehensive culture results from arterial blood, venous blood, and abscess specimens. [file DEO2-6-e70325-s005.docx]

| **Arterial and Venous Blood Culture** | | | | | | |
| --- | --- | --- | --- | --- | --- | --- |
|  |  | |  |  | |  |
| **Gram stain / Smear microscopy** | | | | | | |
| **Category** | | **Finding** | | | **Semi-quant** | |
| Gram stain (smear) | | G+ Rods | | | 1+ | |
|  |  | |  |  | |  |
| **Culture identification** | | | | | | |
| **No.** | | **Organism / Test** | | | **Semi-quant / Result** | |
| 1 | | Clostridium butyricum | | | 1+ | |
|  |  | |  |  | |  |
|  |  | |  |  | |  |
|  |  | |  |  | |  |
| **Abscess Culture** | | | | | | |
|  |  | |  |  | |  |
| **Gram stain / Smear microscopy** | | | | | | |
| **Category** | | **Finding** | | | **Semi-quant** | |
| Gram stain (smear) | | G+ Cocci | | | <+ | |
|  |  | |  |  | |  |
| **Culture identification** | | | | | | |
| **No.** | | **Organism / Test** | | | **Semi-quant / Result** | |
| 1 | | Streptococcus anginosus/milleri | | | 1+ | |
| 2 | | Candida tropicalis | | | <+ | |
| 3 | | Streptococcus constellatus/milleri | | | <+ | |
| 4 | | Bacillus subtilis | | | <+ | |
| 5 | | Prevotella buccae | | | 1+ | |
| 6 | | Lactobacillus sp | | | <+ | |

**Supporting Table S2**

**Comprehensive Culture Results From Arterial Blood, Venous Blood, and Abscess Specimens**
